# A study of the difference in biochemical metabolism between patients with unilateral and bilateral upper urinary tract stones

**DOI:** 10.1038/s41598-024-81454-3

**Published:** 2024-12-03

**Authors:** Zhibin Zheng, Weiguo Hu, Chaoyue Ji, Xuming Zhang, Xijie Ding, Shaobo Zhou, Jianxing Li, Guojun Chen

**Affiliations:** 1 Department of Urology, Beijing Tsinghua Changgung Hospital, School of Clinical Medicine, Tsinghua University, Beijing, 102218 China; 2 Department of Urology, Qinghai University Affiliated Hospital, Xining, 810000 China

**Keywords:** Urinary stones, Bilateral stone, Stone compositions, 24-hour urine, Metabolic analysis, Renal calculi, Nephrology

## Abstract

Bilateral upper urinary tract stones are more likely to lead to impairment of renal function, but few biochemical metabolic studies of bilateral upper urinary tract stones have been reported. We collected clinical data from 555 patients with upper urinary tract stones admitted to Beijing Tsinghua Changgung Hospital from June 2020 to June 2024, and divided them into unilateral and bilateral stone groups by CT scans, analysed the metabolic differences between unilateral and bilateral stone groups by statistical methods, and used multifactorial logistic regression analysis to explore the risk factors that might affect the formation of bilateral stones. A total of 281 cases of unilateral and 274 cases of bilateral stones were identified. The proportion of male patients in the bilateral group was higher than that in the unilateral group (*P* < 0.05). The most prevalent major stone component was calcium oxalate monohydrate (48.1%), with a significantly higher prevalence of cystine stones observed in the bilateral stone group (1.8%) compared to the unilateral stone group (0.4%) (*P* < 0.05). Blood uric acid, blood BUN, blood creatinine, urine pH, and 24-hour urine output were higher in the bilateral stone group than in the unilateral group (*P* < 0.05). The most prevalent metabolic abnormality was low urine volume (45.7%). Bilateral stone group had higher proportion of patients with hyperuricemia (*P* < 0.05). The results of the multivariate logistic regression analysis showed that male gender (OR 1.489, 95% CI 1.028–2.157) and hyperuricemia (OR 1.662, 95% CI 1.113–2.482) were associated with an increased risk of bilateral stone formation (*P* < 0.05). There are significant differences in biochemical metabolism between unilateral and bilateral upper urinary tract stones. The most common metabolic abnormality in patients with urolithiasis is low urine output, and aggressive water intake is effective in preventing stone formation. For patients with hyperuricemia, a strict dietary regimen is imperative to mitigate the likelihood of bilateral stone formation.

## Introduction

Urolithiasis is a prevalent condition affecting the urinary system. In recent years, improvements in quality of life and dietary habits have contributed to an elevated incidence of stones in the urinary tract. The prevalence of urolithiasis in France is estimated to be approximately 10%, while in China, it is approximately 7.54%^1,2^. In consequence of these factors, the financial burden associated with stone treatment has increased considerably. The United States spends an estimated $2 billion each year on stone management, which further contributes to the financial strain on local healthcare systems.

Urological stones are not only highly prevalent but also have a high recurrence rate. In a study conducted by Eisner et al.^3^, it was observed that 50% of patients who had undergone surgery for the removal of stones experienced a recurrence of stones between a period of 5 to 10 years. This highlights the growing importance of further investigating the underlying causes and mechanisms that contribute to their formation^4,5^. Bilateral upper urinary tract stones are more likely to result in damage to kidney function and are also more challenging to treat than unilateral upper urinary tract stones^6^. While a substantial body of research exists concerning the compositional and metabolic analyses of urinary stones, relatively little attention has been directed toward elucidating the biochemical and metabolic discrepancies between unilateral and bilateral upper urinary tract stones.

A retrospective analysis was therefore performed to summarize the metabolic differences between patients with unilateral and bilateral upper urinary tract stones in terms of baseline data, serum biochemistry, 24-hour urine, and stone compositions. Additionally, this study examines the risk factors associated with the formation of bilateral upper urinary tract stones, with the objective of providing a comprehensive framework for the management and prevention of this condition.

## Materials and methods

### Study patients

The research proposal was approved by the Research Ethics Committee of Tsinghua Changgung Hospital at Tsinghua University. This study analyzed 555 patients with upper urinary tract stones at Beijing Tsinghua Changgung Hospital from June 2020 to June 2024. All patients were diagnosed with kidney stones or ureteral stones by CT scans and had complete information about their medical appointments. All patients provided informed consent and participated in the study. The following criteria were utilized to determine the exclusion of participants from the study: Patients with a history of urinary surgeries, congenital or acquired isolated kidneys, medullary sponge kidneys (MSK), renal tubular acidosis (RTA), renal malignancy, primary hyperoxaluria, hypercystinuria, urinary tract abnormalities (including ureteral stenosis, duplicated kidney, horseshoe kidney), neurogenic bladders, or in combination with lower urinary tract stones, including bladder stones and urethral stones, are eligible for exclusion in the study.

### Stone composition analysis and classification

All stones were obtained via percutaneous nephrolithotomy (PCNL) and retrograde intrarenal surgery (RIRS). Of the total number of patients with stones, 492 (88.6%) underwent PCNL, while 63 (11.4%) underwent RIRS. In accordance with the prescribed procedure, the stones were promptly conveyed for immediate examination. The material was ultimately subjected to Fourier transform infrared spectroscopy (FTIR) in order to ascertain its chemical composition. The stone type was defined on the basis of the primary stone components, which constituted a minimum of 50% of the total^7^.

### Diagnostic methods

Baseline information (gender, age), blood pressure, height, weight, past medical history (hypertension, diabetes mellitus), laboratory information (serum biochemistry, 24-hour urine, stone compositions) and relevant diagnostic imaging data were collected and collated. Hypertension is diagnosed based on the following criteria: A systolic blood pressure exceeding 130 mmHg and/or a diastolic blood pressure above 85 mmHg; a documented history of hypertension; or a history of receiving antihypertensive pharmacotherapy^8^. The diagnosis of diabetes mellitus is based on a fasting blood glucose (FBG) > 6.1 mmol/L or 2-hour postprandial glucose (2hPG) > 7.8 mmol/L, or diagnosed and treated^9^. Hyperuricemia is diagnosed when the blood uric acid level is > 420 µmol/L^10^. In accordance with the diagnostic criteria established by the World Health Organization (WHO), obesity is defined as a condition that is identified when the subject’s body mass index (BMI) is found to be equal to or in excess of 30 kg/m²^11^. In accordance with the reference range of our center’s test indicators, a diagnosis of hypercalcemia was rendered when the blood calcium level was > 2.52 mmol/L, and a diagnosis of high PTH was rendered when the PTH was ≥ 65 ng/L. The 24 h urine metabolism abnormality standard is defined in accordance with the urinary stone guideline formulated by the European Association of Urology (EAU)^7^: 24 h urine volume < 2000 ml is low urine output, 24 h urine calcium > 7.5mmol is hypercalciuria, 24 h urine sodium > 260mmol is hypernatriuria, 24 h urine phosphorus > 24.8mmol is hyperphosphaturia, 24 h uric acid > 4425umol is hyperuricosuria, and hypomagnesiuria was defined as 24 h urinary magnesium < 2.46mmol. The classification of unilateral and bilateral upper urinary tract stone groups was based on the results of CT scanning. A comparison was conducted between the two groups with respect to general information, comorbidities, and metabolic differences, and an investigation was undertaken into the risk factors for bilateral upper urinary tract stones.

### Statistical analyses

Statistical analysis was performed using SPSS 25.0 software. Measurement data was represented as X ± s, and counting data was represented as a rate (%). Inter-group comparisons were conducted using Chi-squared tests, T-tests and Fisher’s exact test. A multifactorial logistic regression analysis was performed with the objective of investigating the factors associated with the formation of bilateral upper urinary tract stones. For the purposes of this analysis, the grouping of stones was used as the dependent variable (unilateral group = 0, bilateral group = 1). A p-value of *p* < 0.05 indicates a statistically significant difference.

## Results

### General data characteristics of patients in the unilateral and bilateral groups

A total of 555 patients with upper urinary tract stones were included in this study, comprising 323 males (58.2%) and 232 females (41.8%). The ratio of males to females was 1.39:1, with a mean age of 51.9 ± 14.2 years. Of the total number of urinary stones, 281 (50.6%) were unilateral and 274 (49.4%) were bilateral. The proportion of male patients in the bilateral stone group (31.0%) was markedly elevated in comparison to the unilateral stone group (27.2%) (*P* < 0.05). Additionally, the diastolic blood pressure level of the patients in the bilateral stone group was significantly higher than that in the unilateral stone group (*P* < 0.05). While the BMI level of the patients in the unilateral group was also markedly elevated in comparison to that of the bilateral group (*P* < 0.05). The two groups exhibited no statistically significant discrepancy in age or systolic blood pressure levels (*P* > 0.05) (Table 1).

**Table 1:** General data characteristics of patients with unilateral and bilateral groups.

General information	Unilateral	Bilateral	$\:x$^2^/t	*P* Value
N	281	274		
Gender			4.657	0.031
Male	151(27.2)	172(31.0)		
Female	130(23.4)	102(18.4)		
Age	52.11 ± 14.25	51.68 ± 14.10	0.019	0.718
Blood pressure				
Systolic blood pressure	134.59 ± 18.47	137.11 ± 18.21	0.200	0.107
Diastolic blood pressure	78.80 ± 10.25	81.62 ± 11.25	2.550	0.002
BMI(kg/m^2^)	25.61 ± 4.46	24.77 ± 4.12	0.001	0.021

## The characteristics of the major stone components in the unilateral and bilateral groups

In patients with upper urinary tract stones, the most prevalent major stone component was calcium oxalate monohydrate (48.1%), followed by magnesium ammonium phosphate hexahydrate (25.0%) and apatite carbonate (11.1%). Additionally, the analysis of data revealed a statistically significant higher prevalence of cystine stones in the bilateral stone group (1.8%) compared to the unilateral stone group (0.4%) (*P* < 0.05) (Table 2).

**Table 2:** The characteristics of the major stone components in the unilateral and bilateral groups.

Main component	Group			$\:x$^2^/Fisher	*P* value
	Total (*n* = 555)	Unilateral (*n* = 281)	Bilateral (*n* = 274)		
COM	267(48.1)	146(26.3)	121(21.8)	3.378	0.066
MAP	139(25.0)	63(11.4)	76(13.7)	2.089	0.148
CA	62(11.1)	32(5.8)	30(5.3)	0.027	0.870
UA	56(10.1)	29(5.2)	27(4.9)	0.033	0.855
COD	17(3.1)	8(1.5)	9(1.6)	0.090	0.765
CYS	12(2.2)	2(0.4)	10(1.8)	-	0.020
AAU	1(0.2)	1(0.2)	0	-	-
CaHP	1(0.2)	0	1(0.2)	-	-

## Metabolic characteristics of patients in the unilateral and bilateral groups

In terms of serum biochemistry, the levels of serum magnesium and estimated glomerular filtration rate (eGFR) in the unilateral group were observed to be higher compared to those in the bilateral group (*P* < 0.05). However, the levels of serum uric acid, blood BUN, blood creatinine, and urine pH were lower than those in the bilateral group (*P* < 0.05). Blood calcium, blood sodium and PTH were not statistically significant (*p* > 0.05). Additionally, the 24-hour urinary calcium level in the unilateral group was higher than that in the bilateral group, while the urinary volume was lower than that in the bilateral group (*P* < 0.05). The two groups exhibited no statistically significant discrepancy in urinary sodium, urinary potassium, urinary phosphorus, urinary chloride and urinary uric acid (*P* > 0.05) (Table 3).

**Table 3:** Metabolic characteristics of patients with unilateral and bilateral groups.

Metabolic index	Group		*P* value
	Unilateral(*n* = 281)	Bilateral (*n* = 274)	
Serum biochemical			
Calcium (mmol/L)	2.29 ± 0.12	2.28 ± 0.14	0.939
Magnesium (mmol/L)	0.87 ± 0.07	0.86 ± 0.09	0.012
Sodium (mmol/L)	141.75 ± 2.30	142.06 ± 2.28	0.112
Uric acid (µmol/L)	352.84 ± 101.54	387.44 ± 108.82	< 0.001
PTH(ng/mL)	56.13 ± 53.46	64.77 ± 60.07	0.074
BUN(mmol/L)	5.66 ± 2.19	7.49 ± 4.92	< 0.001
Creatinine (µmol/L)	78.58 ± 42.90	122.29 ± 113.51	< 0.001
eGFR(mL/min)	92.01 ± 25.87	73.45 ± 32.52	< 0.001
pH (urine)	6.15 ± 0.61	6.26 ± 0.67	0.037
24-hour urine			
Calcium (mmol /d)	4.34 ± 2.66	3.55 ± 2.26	< 0.001
Sodium (mmol/d)	150.46 ± 72.17	152.71 ± 71.41	0.713
Potassium (mmol/d)	30.51 ± 13.18	29.79 ± 12.75	0.511
Phosphorus (mmol /d)	17.09 ± 8.48	16.84 ± 7.81	0.715
Chlorine (mmol/d)	126.80 ± 62.17	127.50 ± 60.59	0.894
Uric acid (µmol d)	2910.11 ± 1161.51	2719.44 ± 1180.09	0.056
Urine volume (L)	1.95 ± 0.81	2.12 ± 0.82	0.015

The most prevalent metabolic abnormality among patients with upper urinary tract stones was low urine output, occurring in 45.7% of cases. Hypertension was the most common comorbid condition, observed in 39.4% of patients. The prevalence of diabetes mellitus, hypercalciuria, and hyperuricosuria was significantly higher in the unilateral stone group compared to the bilateral stone group. Conversely, the incidence of hyperuricemia was considerably higher in the group with bilateral stones compared to those with unilateral stones (*p* < 0.05) (Table 4).

**Table 4:** Characteristics of metabolic abnormalities in patients with unilateral versus bilateral stone groups.

	Total	Unilateral	Bilateral	$\:x$^2^	*P* Value
Diabetes	99(17.8)	60(10.8)	39(7.0)	4.797	0.029
Hypertension	219(39.4)	114(20.5)	105(18.9)	0.294	0.588
Obesity	63(11.3)	35(6.3)	28(5.0)	0.690	0.406
Hypercalcemia	13(2.4)	6(1.1)	7(1.3)	0.107	0.744
Hyperuricemia	162(29.2)	65(11.7)	97(17.5)	10.105	0.001
High PTH	129(23.2)	59(10.6)	70(12.6)	1.611	0.204
Low urine output	254(45.7)	140(25.2)	114(20.5)	3.773	0.052
Hypercalciuria	45(8.1)	31(5.6)	14(2.5)	6.531	0.011
Hyperuricosuria	50(9.0)	32(5.8)	18(3.2)	3.929	0.047
Hypernatriuria	40(7.2)	19(3.4)	21(3.8)	0.169	0.681

### Multivariate logistic regression analysis of factors affecting bilateral stone formation

By including the variables of gender (male), age, diabetes, hypertension, obesity, hypercalcemia, hyperuricemia, high PTH, low urine output, hypercalciuria, and hyperuricosuria in a multifactorial logistic regression analysis. The results showed that male gender (OR 1.489, 95% CI 1.028–2.157) and hyperuricemia (OR 1.662, 95% CI 1.113–2.482) were associated with an increased risk of bilateral stone formation (*P* < 0.05). In contrast, the occurrence of low urine output, hypercalciuria, and hyperuricosuria was identified as an underlying risk factor for the development of unilateral stone formation, with statistical significance (*P* < 0.05) (Table 5).

**Table 5:** Multivariate logistic regression analysis of factors affecting bilateral stone formation.

Parameter	Regression coefficient	Standard error	Wald	*P* value	OR (95%CI)
Gender (male)	0.398	0.189	4.431	0.035	1.489(1.028,2.157)
Age	-0.001	0.007	0.016	0.900	0.999(0.986,1.012)
Diabetes	-0.471	0.243	3.752	0.053	0.624(0.387,1.006)
Hypertension	-0.030	0.197	0.024	0.878	0.970(0.660,1.427)
Obesity	-0.224	0.292	0.589	0.443	0.799(0.451,1.417)
Hypercalcemia	0.091	0.589	0.024	0.877	1.096(0.346,3.475)
Hyperuricemia	0.508	0.205	6.162	0.013	1.662(1.113,2.482)
High PTH	0.188	0.216	0.753	0.386	1.206(0.790,1.842)
Low urine output	-0.467	0.184	6.435	0.011	0.627(0.437,0.899)
Hypercalciuria	-0.889	0.361	6.055	0.014	0.411(0.203,0.835)
Hyperuricosuria	-0.774	0.352	4.851	0.028	0.461(0.231,0.918)
Hypernatriuria	0.411	0.393	1.093	0.296	1.508(0.698,3.256)

## Discussion

Urolithiasis has historically been a global concern in the field of urology. In recent years, epidemiological surveys have documented a year-on-year increase in the prevalence of urinary stones. In the United States, for instance, it is estimated that approximately one in 11 individuals will experience urolithiasis-related discomfort at some point in their lifetime^12^. In a recent cross-sectional study conducted in China, the prevalence of urinary stones was determined to be approximately 11.4%^13^. Despite the gradual standardization and improvement of the current treatment of urinary stones, research on such diseases remains in the exploratory stage. Whether researchers study the stone-forming process of urinary stones by constructing biological or non-biological models^14,15^, or explore their pathogenesis from the perspective of cytology (apoptosis, pyroptosis, iron death)^16,17^,they still do not have any effective promotion programme for the pathogenesis or long-term prevention of urinary stones. Bilateral stones have consistently represented a significant challenge for clinicians, as compared to unilateral stones. Patients with bilateral stones not only exhibit greater damage to renal function, but also demonstrate a markedly elevated recurrence rate of stones following surgery^6,18^. Xia et al.^19^ conducted a follow-up study of 657 patients who had undergone stone surgery. Their findings revealed that the recurrence rate for patients in the bilateral stone group (44.1%) was significantly higher than that observed in the unilateral stone group (23.8%). Furthermore, a multifactorial regression analysis indicated that the risk of recurrence in the bilateral stone group was 1.779 times higher than that observed in the unilateral stone group. A total of 555 patients with upper urinary tract stones were included in this study according to strict criteria. The findings revealed significant differences between the unilateral and bilateral groups with respect to general information (gender, blood pressure, BMI), past history (diabetes mellitus, hyperuricemia), serum biochemistry, and 24-h urine. And it has been established that men and hyperuricaemia represent risk factors for the formation of bilateral upper urinary tract stones. This finding may provide a reference strategy for the treatment and prevention of bilateral stones.

The present study revealed a higher prevalence in male patients than in female patients, which is consistent with the findings of the current study^20,21^. Ye et al.^21^ found that the incidence was about twice as high in male patients as in female patients, with calcium oxalate and uric acid stones being more common in male patients and infected stones being more common in female patients. It is possible that dietary habits are a significant contributing factor. The male population is more likely to consume diets that are high in protein and sodium. Excessive intake of these diets has been linked to increased urinary calcium and uric acid excretion, which in turn increases the risk of stone formation^22^. Additionally, sex hormones have been demonstrated to exert influence over the characteristics of stone composition in patients of different sexes^23^. It has been demonstrated that androgen receptor signaling can directly regulate the transcription of hepatic glycolate oxidase, which is responsible for the conversion of glyoxylate to oxalate^24^. An elevated oxalate concentration in urine is conducive to the formation of oxalate stones. Conversely, estrogen has been demonstrated to diminish the formation of oxalate and uric acid stones by prompting an enhanced excretion of inhibitors in the urine. Infectious stones are closely related to urease-producing bacterial infections. The incidence of infectious stones is higher in women due to the physiological and anatomical characteristics of the urethra, which is prone to urinary tract infections. The present study demonstrated that the incidence rate in the unilateral group was 1.03 times higher than that observed in the bilateral group. However, Li et al.^25^ discovered that the prevalence of the unilateral stone group was 1.75 times greater than that of the bilateral stone group. We believe that there are two reasons for this. Primarily, the diagnosis of all patients with urolithiasis in this study was clarified by computed tomography (CT) scan, which has a higher diagnostic value compared to plain radiographic urography or ultrasound^26^; Secondly, it should be noted that a considerable proportion of patients with urinary stones admitted to our center present with more severe illness, which may introduce a degree of selection bias. Our findings indicated that the levels of blood creatinine and BNP were considerably higher in the bilateral group than in the unilateral group. This observation supports the hypothesis that patients with bilateral stones are more prone to impaired renal function, a conclusion that aligns with the findings of previous studies in this area^6^. It seems reasonable to posit that timely intervention in patients with bilateral stones may prove an effective strategy for reducing the incidence of renal insufficiency or other complications^27,28^. Our findings indicate that cystine stones are more frequently observed in bilateral formations. This observation is corroborated by the findings of Sherer et al.^18^, who reported that 66.7% of patients with cystine stones presented with bilateral stone formation. Cystine stones are predominantly associated with high cystinuria, primarily due to the inability of cystine to undergo proper reabsorption in renal tubules, which results in urine supersaturation and stone formation.

The present study revealed a notable elevation in blood uric acid levels among the bilateral patient group in comparison to the unilateral patient group. Additionally, multifactorial analysis indicated that hyperuricemia represents a risk factor for the formation of bilateral upper urinary tract stones. A Korean cohort study has identified a dose-response relationship between elevated blood uric acid levels and an increased risk of kidney stone formation in male patients^29^. In a recent study, Wen et al.^30^ demonstrated that an elevation in blood uric acid levels is associated with a heightened susceptibility to the formation of uric acid stones. Hyperuricemia is a metabolic abnormality resulting from an increase in purine metabolites within the body. Hyperuricemia has been demonstrated to promote stone formation, which may be related to renal injury, an inflammatory response, and heterogeneous nucleation of crystals^31^. Therefore, for patients with hyperuricemia, it is possible to regulate blood uric acid levels through dietary modification or the use of pharmacological agents^32^. In a contrasting study, Narang et al.^33^ discovered an insignificant correlation between uric acid levels and the formation of uric acid stones through an analysis of a UK database comprising biological samples (OR 1.03, 95% CI 0.99–1.08). The results of our study indicate that male patients are at an elevated risk for developing bilateral stones. Additionally, male patients tend to engage in more strenuous forms of exercise, and excessive fluid loss or inadequate water intake can also increase the likelihood of stone formation, which may contribute to this increased risk. The 24-hour urine test reflects the metabolic state of the body and conveys simultaneous information about the urinary microenvironment in which stones grow. Patients with hypercalciuria have an increased risk of developing calcium oxalate stones. Patients with hyperuricosuria are more prone to forming urate stones. Additionally, patients with low urine output are more likely to experience stone formation than the general population^34^. Therefore, making appropriate adjustments according to the results of the 24-hour urine test can be effective in preventing urinary stones^35^. The present study revealed a significantly higher concentration of urinary calcium in the unilateral stone group in comparison to the bilateral stone group. Diets that are high in animal protein and sodium intake have been demonstrated to lead to an increase in urinary calcium excretion. Consequently, modifying unfavorable dietary habits and increasing water intake may prove to be an efficacious strategy for averting stone formation^36,37^. It is recommended that patients with urinary stones consume a daily fluid intake of 2.5 to 3 L. An increase in water intake can result in a dilution of the urinary calcium concentration, thereby reducing the likelihood of the formation of saturated calcium oxalate crystals. In a study conducted by Bernard and colleagues^38^, it was observed that an increase of one liter in daily water intake among adolescent patients resulted in a 710-milliliter increase in urine output over a 24-hour period.

Metabolic syndrome is a disease syndrome that is mainly characterized by abdominal obesity and insulin resistance, with the presence of other metabolic abnormalities, such as hypertension and hyperlipidemia. It is a significant risk factor for the formation and recurrence of urinary stones^39^. Wu et al.^40^discovered that patients with obesity exhibited a heightened propensity for stone formation relative to those with a normal body mass index (BMI). Furthermore, the risk was markedly elevated when metabolic syndrome was present. The presence of obesity has been demonstrated to contribute to the promotion of chronic, systemic inflammation and oxidative stress. This is achieved through the increase in expression of specific adipokines, in addition to the alteration of inflammatory molecules. The latter of these two processes has been shown to play a role in the formation of kidney stones. The aforementioned processes are known to be influenced by the presence of specific inflammatory factors, most notable of which are interleukin-6 (IL-6) and tumor necrosis factor (TNF)^41^. Furthermore, the metabolic syndrome, which is characterized by essential hypertension, diabetes, and obesity, has been demonstrated to promote the excretion of metabolites such as calcium, oxalic acid, and uric acid in the urine, thereby increasing the risk of developing urinary stones^42^. Elevated blood uric acid and fasting blood glucose levels are both significant contributors to the development of the metabolic syndrome and are linked to an increased risk of urinary stone formation^39,43^. A retrospective clinical study revealed a 21.967-fold increase in the probability of bilateral staghorn stones when the metabolic syndrome component parameter was increased from 0 to 3–4. Therefore, it can be inferred that patients with a greater number of metabolic parameters are at a higher risk of developing stones^44^. In a separate study, Li et al.^25^ found that patients diagnosed with metabolic syndrome and presenting with five of the identified parameters exhibited a 1.89-fold elevated risk of developing bilateral upper urinary tract stones relative to patients exhibiting only a single parameter(OR 3.381 95%CI 1.221–9.360;*P* = 0.013). In the present study, we observed that patients with unilateral stones demonstrated a higher prevalence of comorbid diabetes mellitus, whereas multifactorial analysis revealed that diabetes mellitus did not constitute a risk factor in the group with bilateral upper urinary tract stones. This may be attributable to the relatively low number of metabolic syndrome parameters.

In our study, low urine output and hypercalciuria were associated with a reduced risk of bilateral stone formation, but with an increased risk of unilateral stone formation. We carefully reviewed the past literature, which is inconsistent with previous literature^25^. Therefore, we consider that bilateral renal haemodynamics may be different, which could potentially give rise to variations in glomerular filtration, fluid concentrations, and subsequent risk of stone formation. In a prospective study conducted by Shekarriz et al.^45^, a correlation was identified between stone formation and sleeping position. Of the 93 patients who experienced stone recurrence and slept in a side-dependent position, 76% had kidney stones on the side they habitually slept on (*p* = 0.008). Additionally, Ziaee et al.^46^ demonstrated a notable correlation between the lateral position during sleep and the development of kidney stones in the corresponding kidney. A review of the sleep positions of 38 patients with recurrent unilateral kidney stones, as recorded by a sleep monitor, revealed that 88.2% of the 17 patients with right kidney stones exhibited a right-side-down dependent sleep position, while 61.9% of the 21 patients with left kidney stones demonstrated a left-side-down dependent sleep position. Low urine output or hypercalciuria represents an intrinsic risk factor for the formation of stones^34^. It is possible that maintaining a sleep-dependent position on one side may also increase the risk of unilateral stones. Furthermore, it is important to consider the limitations of single-centre studies. It may be beneficial to conduct multi-centre studies to gain a more comprehensive understanding of the validation of this idea.

This study employs a retrospective clinical case analysis to investigate the pathogenesis and risk factors associated with unilateral and bilateral upper urinary tract stones. It considers general data, serum biochemistry, and 24-hour urine, with the aim of providing insights that may inform the prevention of bilateral stone formation. However, as this study is a single-center study, it is unable to reflect stone formation in different geographical areas and different races. This may result in selection bias, and a series of prospective or multicenter studies are required to further explore the authentication in the follow-up. Furthermore, our center does not currently perform 24-hour urine pH, citrate, and oxalate indices, which may contribute to some of the imperfections identified in the 24-hour urine study. Notwithstanding these constraints, our investigation furnished clinical urologists and medical practitioners with empirical data, thereby facilitating the formulation of more effective prevention protocols for individuals at heightened risk of developing bilateral upper urinary tract stones.

## Conclusion

The present study indicates that there are significant differences in biochemical metabolism between unilateral and bilateral upper urinary tract stones. The most frequently observed metabolic abnormality in individuals with urinary calculi is low urine output. Consumption of sufficient quantities of water can effectively prevent the formation of kidney stones. Additionally, hyperuricemia has been identified as a contributing factor to the development of bilateral stones. Consequently, for patients with hyperuricemia, a strict dietary regimen is imperative to mitigate the likelihood of bilateral stone formation.

## Data Availability

The data used or analyzed in this study are available from the corresponding author upon reasonable request.

## Abbreviations

eGFR: Estimated glomerular filtration rate
PTH: Parathyroid hormone
BUN: Urea nitrogen
CT: Computerized tomography
BMI: Body mass index
MSK: Medullary sponge kidneys
RTA: Renal tubular acidosis
FBG: Fasting blood glucose
COD: Calcium oxalate dihydrate
COM: Calcium oxalate monohydrate
UA: Uric acid
MAP: Magnesium ammonium phosphate hexahydrate
AAU: Ammonium acid urate
CYS: Cystine
CA: Carbonate apatite
CaHP: Calcium dihydrogen phosphate
